# Ultrastructural studies of the neurovascular unit reveal enhanced endothelial transcytosis in hyperglycemia‐enhanced hemorrhagic transformation after stroke

**DOI:** 10.1111/cns.13571

**Published:** 2021-01-01

**Authors:** Xiaomin Xu, Liuqi Zhu, Ke Xue, Jiayi Liu, Jian Wang, Guohua Wang, Jin‐hua Gu, Yunfeng Zhang, Xia Li

**Affiliations:** ^1^ Institute of Special Environmental Medicine Co‐innovation Center of Neuroregeneration Nantong University Nantong China; ^2^ Department of Neurology Affiliated Hospital of Nantong University Nantong China; ^3^ Qidong Women's and Children's Health Qidong China

**Keywords:** blood, brain barrier, electron microscopy, endothelial vesicles, hyperglycemia, neurovascular unit

## Abstract

**Aims:**

Pre‐existing hyperglycemia (HG) aggravates the breakdown of blood–brain barrier (BBB) and increases the risk of hemorrhagic transformation (HT) after acute ischemic stroke in both animal models and patients. To date, HG‐induced ultrastructural changes of brain microvascular endothelial cells (BMECs) and the mechanisms underlying HG‐enhanced HT after ischemic stroke are poorly understood.

**Methods:**

We used a mouse model of mild brain ischemia/reperfusion to investigate HG‐induced ultrastructural changes of BMECs that contribute to the impairment of BBB integrity after stroke. Adult male mice received systemic glucose administration 15 min before middle cerebral artery occlusion (MCAO) for 20 min. Ultrastructural characteristics of BMECs were evaluated using two‐dimensional and three‐dimensional electron microscopy and quantitatively analyzed.

**Results:**

Mice with acute HG had exacerbated BBB disruption and larger brain infarcts compared to mice with normoglycemia (NG) after MCAO and 4 h of reperfusion, as assessed by brain extravasation of the Evans blue dye and microtubule‐associated protein 2 immunostaining. Electron microscopy further revealed that HG mice had more endothelial vesicles in the striatal neurovascular unit than NG mice, which may account for their deterioration of BBB impairment. In contrast with enhanced endothelial transcytosis, paracellular tight junction ultrastructure was not disrupted after this mild ischemia/reperfusion insult or altered upon HG. Consistent with the observed increase of endothelial vesicles, transcytosis‐related proteins caveolin‐1, clathrin, and hypoxia‐inducible factor (HIF)‐1α were upregulated by HG after MCAO and reperfusion.

**Conclusion:**

Our study provides solid structural evidence to understand the role of endothelial transcytosis in HG‐elicited BBB hyperpermeability. Enhanced transcytosis occurs prior to the physical breakdown of BMECs and is a promising therapeutic target to preserve BBB integrity.

## Graphical abstract

Ultrastructural analysis of the blood–brain barrier (BBB) after mild ischemic stroke reveals that acute hyperglycemia (HG) increases endothelial vesicles but does not alter tight junction structure. Enhanced endothelial transcytosis occurs prior to the physical breakdown of endothelial cells, thereby contributing to post‐stroke BBB hyperpermeability under HG condition.

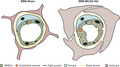

## 1 INTRODUCTION

Hyperglycemia (HG) is a highly prevalent comorbid condition in acute ischemic stroke patients and can be a chronic condition persisting for days.^1^ HG is associated with larger infarct size, more severe brain edema, poorer clinical outcome, and a higher risk of mortality and hemorrhagic transformation (HT). The mechanisms underlying increased occurrence of post‐stroke HT under HG condition are not fully understood, but exacerbation of blood–brain barrier (BBB) damage may play a crucial role.^2^, ^3^, ^4^, ^5^, ^6^, ^7^ High blood glucose leads to endothelial cell dysfunction and increased intracellular reactive oxygen species (ROS), which are involved in a plethora of pathophysiological changes such as inhibition of nitric oxide (NO) synthesis, vascular inflammation, insulin resistance, neovascularization, leukocyte adhesion, and protein and polymer glycosylation.^8^, ^9^, ^10^, ^11^, ^12^ Intriguingly, whether the efficacy of drugs targeting endothelial cell dysfunction is a direct result of improving endothelial functions is disputable under different disease conditions and at different vascular sites.^8^, ^13^, ^14^, ^15^, ^16^, ^17^ A thorough understanding of the regulatory mechanisms of endothelial functions under HG condition is warranted to develop effective therapeutic approaches to ameliorate BBB injury.

Brain microvascular endothelial cells (BMECs) are major contributors to the permeability barrier in the neurovascular unit (NVU). BMECs regulate substance exchange at the BBB interface and restrict BBB permeability by forming paracellular barriers that permit limited diffusion of blood‐derived solutes and allowing only sparse endocytic vesicles for transcytosis.^18^, ^19^ Previous studies have shown that enhanced transcellular transport may be the initial response of CNS endothelial cells during stroke.^20^ Uptake and transport of Alexa594‐labeled albumin into the brain parenchyma by vascular endothelial cells occur as early as 6 h after stroke, accompanied by an increase in the number of endocytic vesicles.^20^ That study indicates that the increase in BBB permeability at the early stage after stroke is not due to the degradation of tight junctions, but instead is related to increased endothelial transcytosis. In another subsequent study, ultrastructural analysis also favored the number of endothelial vesicles as the best temporal and spatial indicator of BBB destruction after cerebral ischemia.^21^ A limitation of that study, however, is that it did not investigate the type of the transcytotic vesicles or the underlying mechanisms.

Endocytosis is a cellular process in which extracellular materials are transported into the cell through the deformation and internalization of cell membrane. Depending on the type of materials being taken up by the cell, endocytosis can be classified into phagocytosis and pinocytosis. On the basis of the size of the molecules and receptor involved in the process, pinocytosis is further divided into macropinocytosis (0.2–10 µm) and micropinocytosis (<0.2 µm),^22^ and the micropinocytosis is conveniently classified by the nature of the coat proteins associated with the endocytic process into clathrin‐mediated endocytosis (~150 nm), caveolin‐mediated endocytosis (~100 nm), and clathrin‐ and caveolin‐independent pinocytosis.^23^, ^24^, ^25^ Among them, caveolin‐mediated pinocytosis is a common example of micropinocytosis that is formed in the epithelium of the blood vessels. Although enhanced endothelial endocytosis is known to play a critical role in early BBB injury after stroke,^20^, ^26^, ^27^ the type and cause of increased endocytic vesicles remain unknown. To date, the role of endocytosis in hyperglycemia‐elicited vascular endothelial cell dysfunction is poorly understood, nor are the ultrastructure changes at the BBB interface thoroughly characterized in the post‐stroke brain under HG condition.

In this study, we used two‐ and three‐dimensional reconstruction by electron microscopy to elucidate the morphological and ultrastructural changes at the BBB interface after mild ischemia/reperfusion (I/R) brain injury, aiming to provide reliable structural evidence to understand the potential mechanisms underlying HG‐exacerbated HT.

## 2 MATERIALS AND METHODS

### 2.1 Animals and experimental models

Healthy male ICR mice (8–12 weeks old, 20–22 g) were obtained from the Experimental Animal Center of Nantong University. Mice were housed with a 12/12‐hour light/dark cycle and bred with ad libitum access to food and water. All the processes of animal experiments were approved by the Institutional Animal Care and Use Committee of Nantong University.

Mice were randomly assigned to three groups: sham control group, middle cerebral artery occlusion (MCAO) group with normoglycemia (MCAO‐NG), and MCAO group with hyperglycemia (MCAO‐HG). Mice in the MCAO‐HG group received intraperitoneal injection of 50% glucose (0.06 mL/10 g) 15 min before brain ischemia to induce acute hyperglycemia.^28^, ^29^ Mice were anesthetized by isoflurane, and a skin incision was made to expose the right common carotid artery (CCA), external carotid artery (ECA), and internal carotid artery (ICA). A 6–0 monofilament with silicon‐coated tip (6023PK5Re, Doccol Corporation) was introduced from the ECA to the ICA until it reached the beginning of the MCA. After 20 min of MCA occlusion, the filament was removed from the vessel lumen. Sham‐operated mice had the same anesthesia and surgical procedures except the insertion of the filament. Cerebral blood flow was monitored throughout the surgery using laser Doppler flowmetry. After 4 h of post‐MCAO reperfusion, the mice were euthanized for the following experiments.

### 2.2 Assessment of BBB permeability

After 3 h of post‐MCAO reperfusion, 2% Evans Blue (0.1 mL/10 g) was injected through tail vein. One hour later, mice were anesthetized with intraperitoneal injection of 2.5% avertin (2,2,2‐tribromoethanol, Sigma‐Aldrich, 400 mg/kg body weight) and transcardially perfused with 0.9% NaCl and 4% paraformaldehyde. Brains were removed, and coronal sections of the brain were cut. After photographs of the sliced brains were captured, brain tissues were fixed in paraformaldehyde again and cryoprotected in 30% sucrose in PBS. Frozen serial coronal brain sections (25 μm thick) were prepared, mounted, and imaged by Leica TSC SP8 confocal microscope (Leica, Germany). The severity of BBB leakage was evaluated by the fluorescence intensity of Evans blue dye in the brain, as measured by the ImageJ software (version 1.43b, NIH, USA).

### 2.3 Immunofluorescence staining

Immunofluorescence staining was performed on 25‐μm‐thick free‐floating brain sections as previously described.^30^ Briefly, brain sections were permeabilized, blocked, and incubated with rabbit anti‐microtubule‐associated protein 2 (MAP2, Cell Signaling Technology, USA) overnight at 4°C. Sections were then washed by PBS and incubated with fluorochrome‐conjugated secondary antibodies. Images were acquired by Nikon Eclipse Ni‐E microscope (Nikon, Japan), and infarct areas were measured on six equally spaced coronal sections (25 μm, take 6 slices for every 10 slices) within the MCA territory per brain by ImageJ software (version 1.43b, NIH, USA).

### 2.4 Assessment of neurological deficits

Double‐blind standard was conducted throughout the test. Neurological deficits were assessed 4 h after reperfusion according to the Clark's scoring system,^31^ including a general neurological scale (0–28) and a focal neurological scale (0–28). Six areas were assessed for the general score, and seven areas were assessed for the focal score to evaluate the severity of neurological deficits after ischemic stroke.

### 2.5 Transmission electron microscopy (TEM)

According to the results of MAP2 immunostaining, tissue blocks (1 mm^3^) from the striatum of the bregma layer of brain tissue were acquired under a stereomicroscope, and fixed in fresh 2.5%GA/0.1 M CAS at 4°C. After 3 times of rinse, tissue blocks were put into 2% OsO_4_ in 2% K_3_Fe(CN)_6_/0.1 M CAS for 2 h at 4°C, protected from light, and rinsed with distilled water. Tissue blocks were then proceeded for 1% uranyl acetate staining for 2 h, protected from light, and rinsed with distilled water. The samples were treated with increasing concentration of ethanol (30%, 50%, 70%, 80%, 90%, 95%, and 100%), each for 20 min, to dehydrate, followed by a mixture of ethanol and acetone (1:1) for 20 min. The samples were then treated with pure acetone (20 min), a mixture of embedding resin (Epon812 kit, Electron Microscopy Science, USA) and acetone (V/V = 1/1 for 1 h and V/V = 3/1 for 3 h), and pure resin overnight. Samples were placed in fresh pure resin, embedded for 2 h, and transferred to a new embedding mold for oven heating at 65°C for 24–48 h. A total of 70 nm sections were prepared from the embedded resin block, and stained with lead citrate solution and uranium peroxide acetate solution for 5 min. Images were acquired by a Tecnai Spirit 120 kV transmission electron microscope (ThermoFisher Scientific, USA).

### 2.6 Quantitative analysis of TEM images

TEM micrographs of brain capillaries were collected from the ipsilateral (ischemic) striatum and quantitatively analyzed using ImageJ software (version 1.43b, NIH, USA). Lumen circularity was calculated using the circularity function in ImageJ software (version 1.43b, NIH, USA), whereby a value of 1 indicates a perfect circle.^21^, ^32^ The length of a tight junction was measured from its starting point to endpoint. Endothelial vesicles were counted and expressed as the number of vesicles per mm^2^ of the total endothelial cell area. To visualize the different sizes of endothelial vesicles, frequency distribution histograms on vesicle diameters were plotted.

### 2.7 Serial block‐face (SBF) EM sample preparation and Focused Ion Beam Scanning Electron Microscopy (FIB‐SEM)

Samples were fixed with 2.5% glutaraldehyde for 24 h at 4°C and rinsed with 0.1 M PBS for 3 times. Samples then underwent the following processing steps, with double distilled water rinsing for 5 times between each step: (1) fixation with osmic acid solution (2% osmic acid: 3% potassium ferricyanide =1:1) at 4°C for 1 h; (2) incubation with 1% thiosemicarbazide for 20 min; (3) fixation with 2% osmic acid for 30 min at room temperature; (4) treatment using aspartic acid solution for 30 min at 60°C; and (5) treatment using 1% uranyl acetate overnight at 4°C. Samples were dehydrated using ethanol gradient in the following sequence, each concentration for 15 min: 20%, 50%, 70%, 90%, 100%, and 100%. Samples were subsequently processed for resin penetration by treatment with 100% acetone (10 min), resin:acetone = 3:7 (5–8 h), resin:acetone = 7:3 (8–12 h), and pure resin overnight. Fresh resin was replaced for 2 times, followed by polymerization at 60°C for more than 48 h.

Resin blocks were carefully trimmed using a Leica EM trimming knife until the surface of black tissue in the block was visible. To select the area of interest, we used a scanning electron microscope (Teneo VS, Thermo Fisher Scientific, USA) with one ultramicrotome in its specimen chamber, which allowed us to trim the resin blocks and acquire the EM images of the sample simultaneously. After finding the area of interest, imaging was performed using a dual beam scanning electron microscope (FIB Helios G3, Thermo Fisher Scientific, USA). The data collection procedure was operated in the serial‐surface view mode with a slice thickness of 5 nm at 30 keV and 0.79 nA. Each serial face was then imaged with a 2 kV acceleration voltage and a current of 0.2 nA in backscatter mode (BSE) with an ICD detector. The image storage resolution was set at 3072 × 2048 pixels with a dwell time of 15 ms per pixel and 4.25 nm per pixel. Approximately 500 slices were obtained, and one whole NVU was contained in the image stack ~4 μm in depth. The image stack was aligned and cropped using the Amira software (version 6.5, Thermo Fisher Scientific, USA). Segmentation and labeling of endothelial cells and vesicles were carried out manually. The surface generation tools in Amira were used to compute surfaces of the structures of interest.

### 2.8 Immunoblotting

Protein was extracted from the striatum and subjected to Western blotting analysis as previously described.^33^ The following primary antibodies were used: anti‐caveolin‐1 (CAV1; 1:1000, Cell Signaling Technology, USA), anti‐hypoxia‐inducible factor 1 alpha (HIF‐1α; 1:500, R&D Systems, USA), anti‐clathrin (1:1000, Santa Cruz Biotechnology, USA), anti‐occludin (1:1000, Proteintech, USA), and anti‐β‐actin (1:10,000, Sigma‐Aldrich, USA). Digital images of protein bands were acquired on a Tanon 5200 Chemiluminescent Imaging System (TanonScience & Technology, China), and quantitative analysis was performed using ImageJ software (Version 1.43b, NIH, USA).

### 2.9 Statistical analysis

Data are expressed as mean ± standard error (mean ± SEM), and statistical analysis was performed by the SPSS 20.0 software. Paired t‐test was used for comparison of blood glucose levels before and after glucose injection. Differences in means among multiple groups were analyzed by one‐way ANOVA, followed by the Fisher's LSD test. Kruskal–wallis test was used to analyze data that do not conform to normal distribution. A *P* value less than 0.05 was considered statistically significant.

## 3 RESULTS

### 3.1 Hyperglycemia aggravates BBB damage and enlarges brain infarct after mild ischemic stroke

**FIGURE 1:**
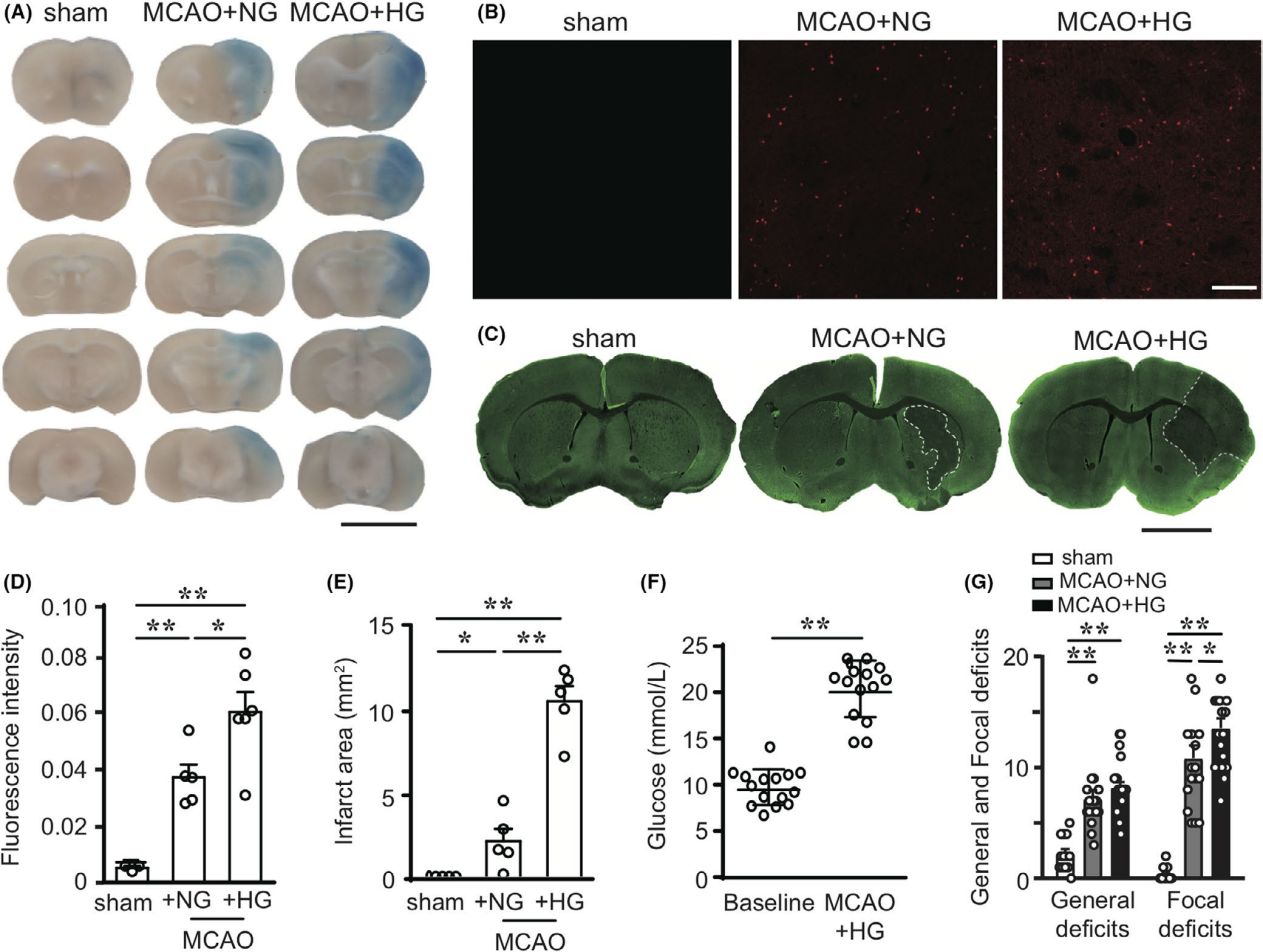
Hyperglycemia exacerbates blood–brain barrier disruption, brain infarct, and neurological deficits after mild ischemic stroke in mice. Mice with normoglycemia (NG) or hyperglycemia (HG) were subjected to mild I/R brain injury induced by 20 min of MCAO and 4 h of reperfusion. (A) Representative images of coronal brain sections demonstrate the extravasation of Evans blue (EB) dye after intravenous injection. Scale bar: 5 mm. (B) Representative images showing the fluorescent signal of EB (red) in the ipsilateral striatum. Scale bar: 100 μm. (C) Representative images of coronal brain sections at bregma 0.5 mm immunostained for MAP2 (green). Brain infarct was delineated with white dashed lines. Scale bar: 2.5 mm. (D) Quantification of EB fluorescence intensity for each group in B (n = 4–5). (E) Quantification of brain infarct size as MAP2‐negative area in C (n = 5). (F) Blood glucose levels were measured before (baseline) and 15 min after intraperitoneal injection of 50% glucose (0.06 mL/10 g, n = 15). (G) Neurological deficits were evaluated in sham‐operated mice and NG and HG mice after mild brain I/R, using the general and focal neurological scales (n = 13–22). Data are mean ± SEM. *P < 0.05, **P < 0.01

To assess BBB integrity after mild I/R (20 min / 4 h) injury, we monitored the extravasation of Evans blue (EB) dye into the brain parenchyma after injection into the mouse tail vein. Similar to previous reports,^34^, ^35^, ^36^ we observed more severe brain leakage of EB after ischemic stroke in the ipsilateral brain tissues of HG mice, compared to NG controls (Figure 1A,B). To further quantitatively evaluate the extent of EB leakage, fluorescence intensity was used to measure the EB absorbance at 620 nm. Both NG and HG mice had dramatically elevated EB fluorescence intensity at 4 h of reperfusion after 20‐min ischemia (Figure 1D), whereby EB fluorescence intensity was approximately 1.5 times higher in the HG group than in the NG group (Figure 1D).

The deterioration of BBB dysfunction was accompanied by enlarged brain infarct in the HG mice after MCAO, as assessed by immunostaining of MAP2, a protein predominantly expressed in the dendrites. After 4 h of reperfusion, brain infarct (MAP2‐negative area) had already formed even in the NG group (Figure 1C). However, brain injury remained mild at this time point and the infarct was limited to the striatum. The MCAO‐HG group, on the other hand, developed a much larger infarct after 4 h of reperfusion which encompassed both the striatum and cortex (Figure 1C,E).

To minimize individual differences among mice, we monitored the blood glucose of each mouse, and only selected those mice with blood glucose higher than 20 mmol/L for the MCAO model (Figure 1F). MCAO/reperfusion induced prominent neurological deficits (on both the general and focal scales) in NG and HG mice, compared to the sham control group (Figure 1G). HG mice demonstrated more severe focal deficits than NG mice, whereas no significant difference was found between HG and NG mice in general deficits after mild stroke (Figure 1G). Together, these results suggested that hyperglycemia increased BBB permeability, and exacerbated brain injury and neurological deficits after mild ischemic stroke.

### 3.2 Hyperglycemia enhances transcellular but not paracellular pathway in BMECs after mild I/R brain injury

To elucidate how hyperglycemia influenced BBB permeability and integrity, we examined the ultrastructure of the NVU by TEM imaging,^37^ focusing on microvessels with a dimeter less than 8 μm. With the guidance of MAP2 immunostaining in adjacent brain sections, we were able to locate the infarct core region in the ipsilateral striatum, which exhibited the most prominent EB leakage.

**FIGURE 2:**
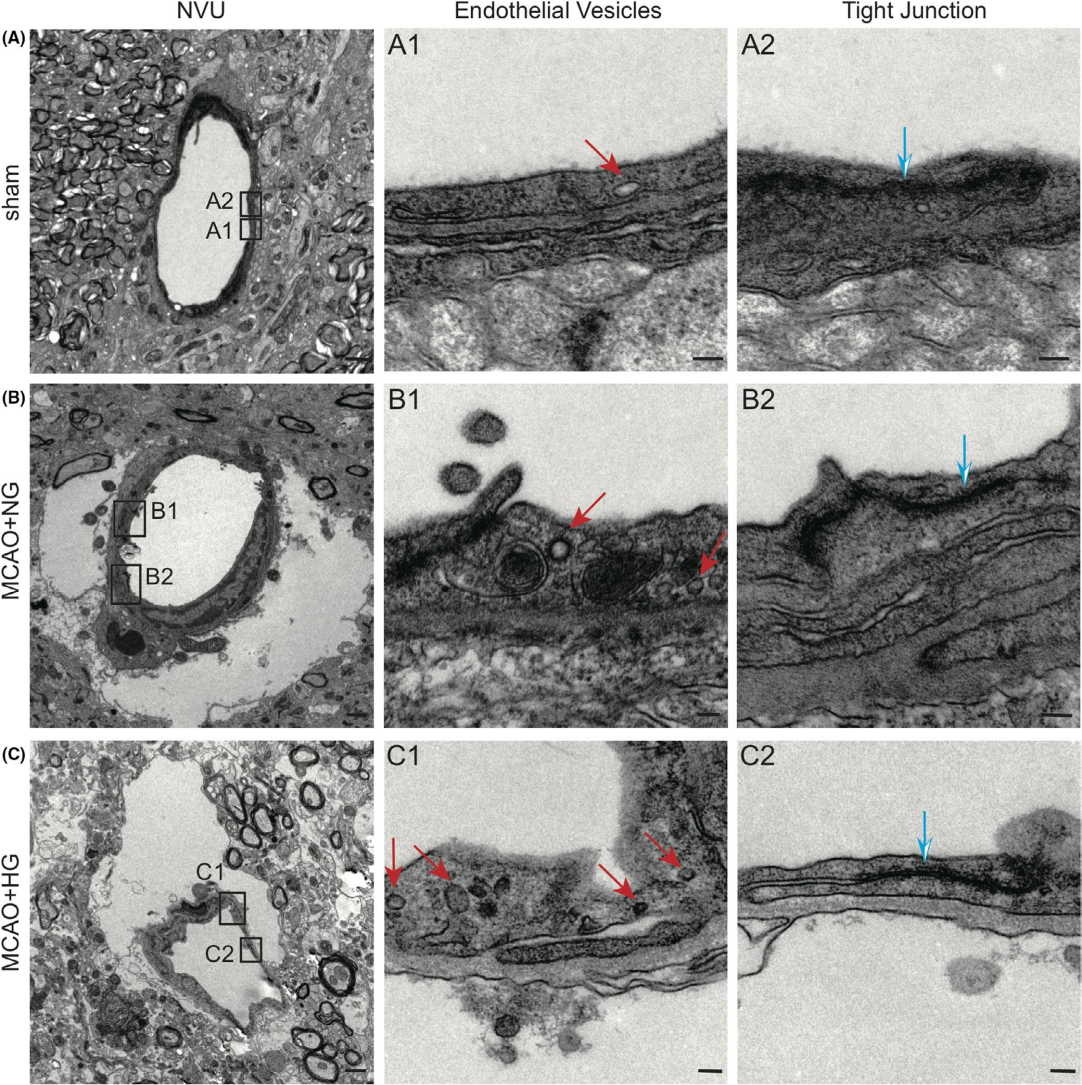
Ultrastructure of the neurovascular unit in the infarct core in the striatum after mild ischemia/reperfusion brain injury. Representative TEM images of the NVU in the striatum of sham‐operated mice (A) and post‐MCAO mice under NG (B) and HG (C) conditions after 20 min of MCAO and 4 h of reperfusion. (A1‐C1) High‐magnification images of the area boxed in (A‐C), highlighting endothelial vesicles with red arrows. (A2‐C2) High‐magnification images of the area boxed in (A‐C), highlighting tight junctions with blue arrows. Scale bars: 1 μm in A‐C, 100 nm in A1‐C1 and A2‐C2

**FIGURE 3:**
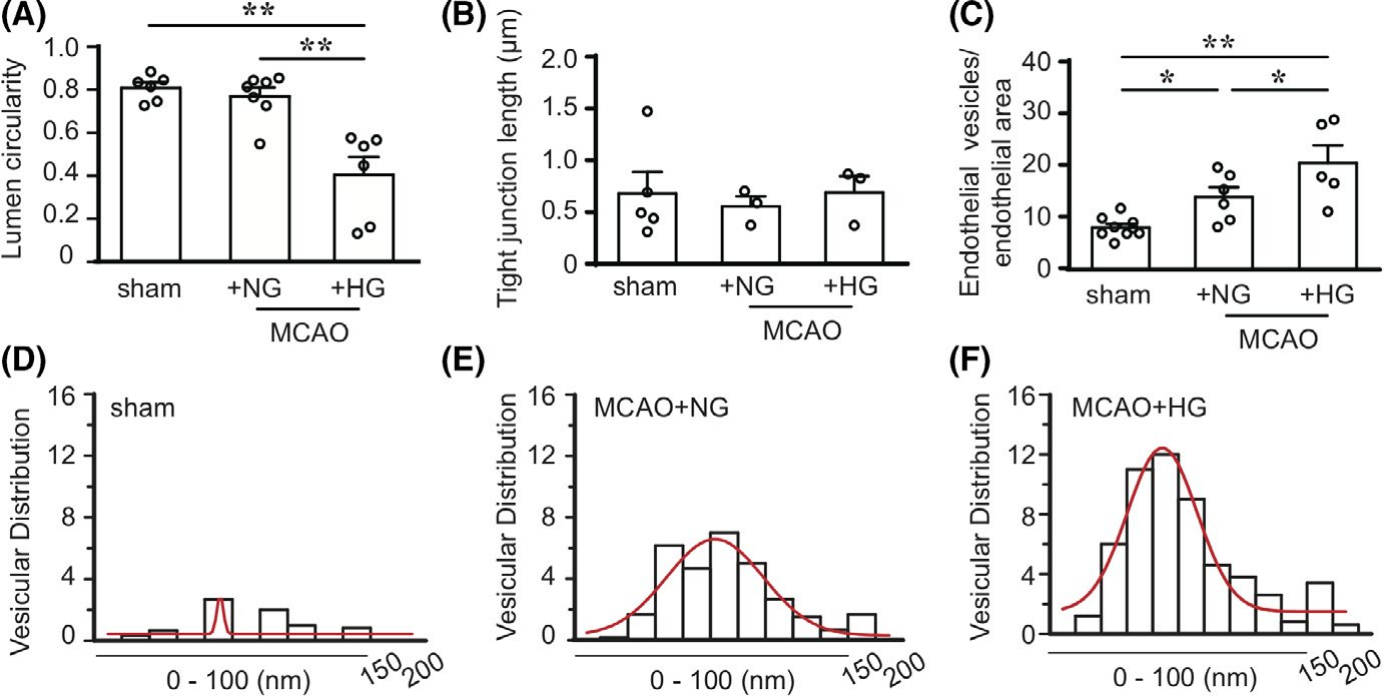
Quantitative analysis of the TEM images on the post‐stroke neurovascular unit. (A‐C) The circularity of lumen (A) (n = 6–7 NVU per group), the length of endothelial tight junctions (B) (Sham: 6 TJs from 5 NVU [6/5]; NG: [5/3]; HG: [5/3]), and the number of endothelial vesicles (C) (Sham: 147 endothelial vesicles from 9 NVU [147/9]; NG: [218/6]; HG: [364/5]) were quantified on TEM images of the striatum 4 h after 20‐min MCAO. D‐F, Frequency distribution of the diameters of endothelial vesicles in sham control group, and in MCAO groups under NG and HG conditions. Data are mean ± SEM, and n values representing the number of TJs, endothelial vesicles, and NVU. *P < 0.05, **P < 0.01

In the sham control group, we observed intact and homogeneous NVU ultrastructure typical for the homeostatic brain,^19^, ^38^, ^39^ characterized by the homogenous intercellular tight junction structures between BMECs, few endothelial vesicles inside BMECs, and surrounding pericytes and astrocyte end‐feet with normal morphology (Figure 2A). The numbers of endothelial vesicles were increased markedly 4 h after MCAO in NG mice (Figure 2B), and were further elevated in the HG group (Figure 2C), whereas no prominent abnormalities were found in the ultrastructure of tight junctions at this time point. Moreover, both NG and HG groups demonstrated intracellular edema after MCAO in the surrounding astrocyte end‐feet and adjacent tissues, manifested by increased clear space within the cytoplasm (Figure 2B,C). Notably, the HG group suffered from prominent collapse of the microvascular wall compared to the NG and sham groups, possibly indicating severe edema under high blood glucose (Figure 2C). This is supported by quantitative analysis of microvessel lumen circularity, wherein the vessels in the HG group had reduced circularity and deformed to a flat or polygon shape under HG condition (Figure 3A).

We further quantified tight junction ultrastructure and endothelial vesicles on TEM images to determine whether post‐stroke extravasation of EB resulted from the paracellular pathway or the transcellular pathway under HG condition. We found that the lengths of tight junctions were similar among all groups (Figure 3B). On the other hand, mild I/R injury significantly increased the numbers of endothelial vesicles normalized to total endothelial area in the NVU at ipsilateral striatum (Figure 3C), indicating enhanced endothelial transcytosis. The HG group demonstrated further increased vesicle numbers compared to the NG group after MCAO/reperfusion. A closer analysis on the distribution of vesicle sizes showed that the diameters of transcellular vesicles ranged from 10 nm to 200 nm in all groups, and vesicle numbers varied in each diameter ranges (Figure 3D‐3F). Compared to the sham group, the stroke groups had dramatically increased numbers of intracellular vesicles in the diameter ranges of 30–80 nm and >100 nm, especially under HG condition (Figure 3D‐F). Collectively, these results suggested that the enhanced transcellular pathway in the post‐stroke NVU in HG mice may involve different types of endocytosis.

### 3.3 Three‐dimensional visualization of endothelial vesicles in the neurovascular unit after mild ischemic stroke

**FIGURE 4:**
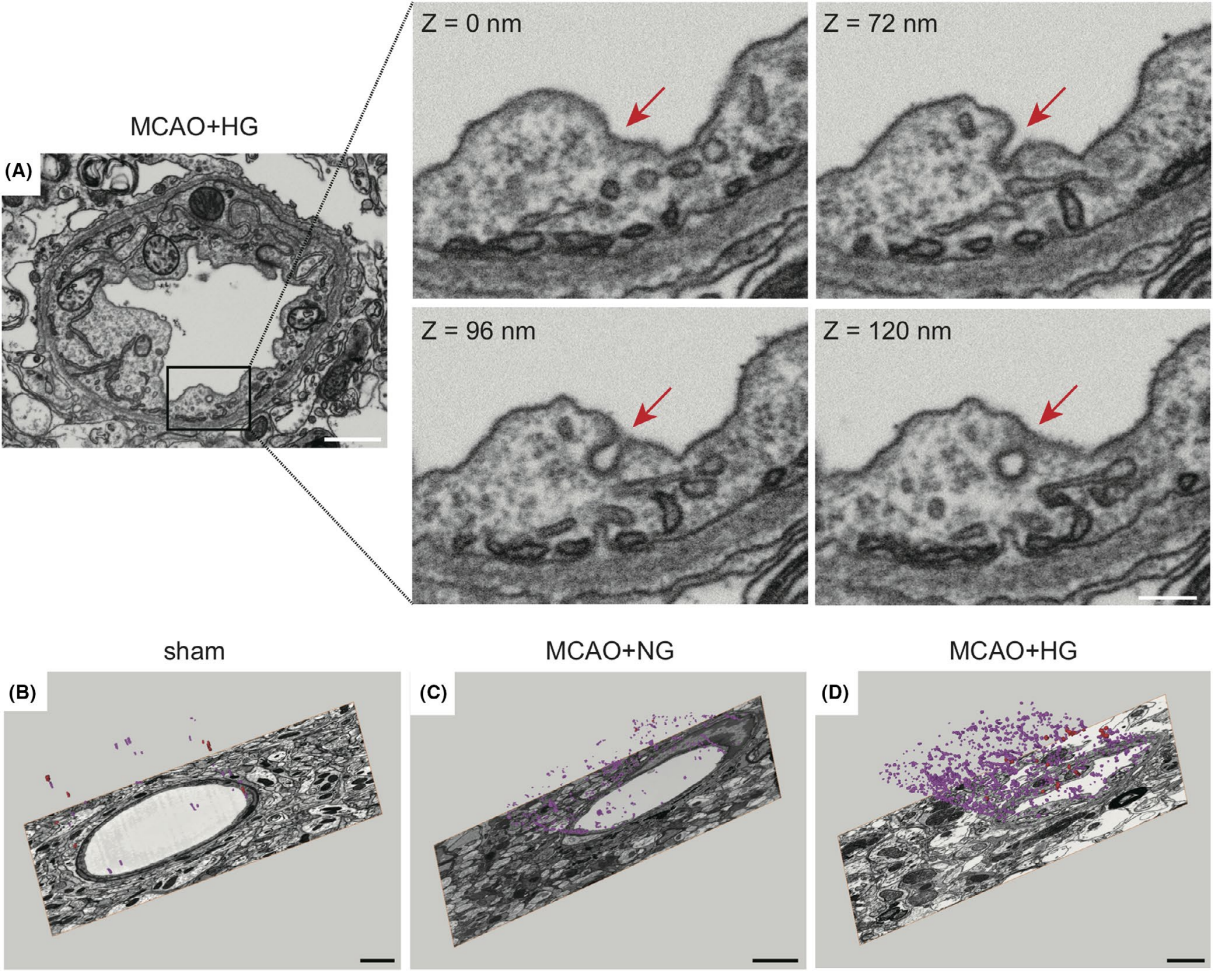
Three‐dimensional reconstruction of the neurovascular unit ultrastructure in the post‐stroke striatum. (A) A representative image slice of the NVU in the hyperglycemia group after MCAO, taken by FIB‐SEM. Red arrows in the right panels indicate a membrane invagination at different z‐axis levels (0, 72, 96, 120 nm). Scale bars: 1 μm (left panel) or 250 nm (middle and right panels). (B‐D) Endothelial vesicles were reconstructed and segmented from a 5‐μm‐thick sample block in each group. Purple denotes the population of vesicles inside endothelial cells of microvessels with a diameter less than 8 μm. Scale bar: 1 μm

Serial block‐free scanning electron microscopy (SBF‐SEM) allows three‐dimensional visualization of large sample volumes at ~50 nm resolution.^40^, ^41^, ^42^ Consistent with the aforementioned two‐dimensional data, compression of microvessels and swelling of surrounding astrocytes were observed in the NVU in the ipsilateral striatum after MCAO under HG condition (Figure 4A). Furthermore, we detected a typical clathrin‐mediated membrane invagination in the ipsilateral NVU, which was coated by lattice‐like densities along the z‐axis (Figure 4A). Non‐coated vesicles of different sizes were also observed in the endothelial cytoplasm (Figure 4A).

To better illustrate the three‐dimensional ultrastructure of the NVU, we imaged about 500 slices from 4‐μm‐thick tissue blocks and segmented the endothelial vesicles in each group. Three‐dimensional surface rendering of segmented endothelial vesicles was performed in Amira software. The reconstructed 3D structures of endothelial vesicle networks were shown in Figure 4B‐D and Movie S1–S3. These results based on large‐scale NVU samples further confirmed that high blood glucose increased endothelial vesicles after stroke.

### 3.4 Hyperglycemia enhances CAV1‐ and clathrin‐mediated endocytosis in brain endothelial cells after mild I/R injury

**FIGURE 5:**
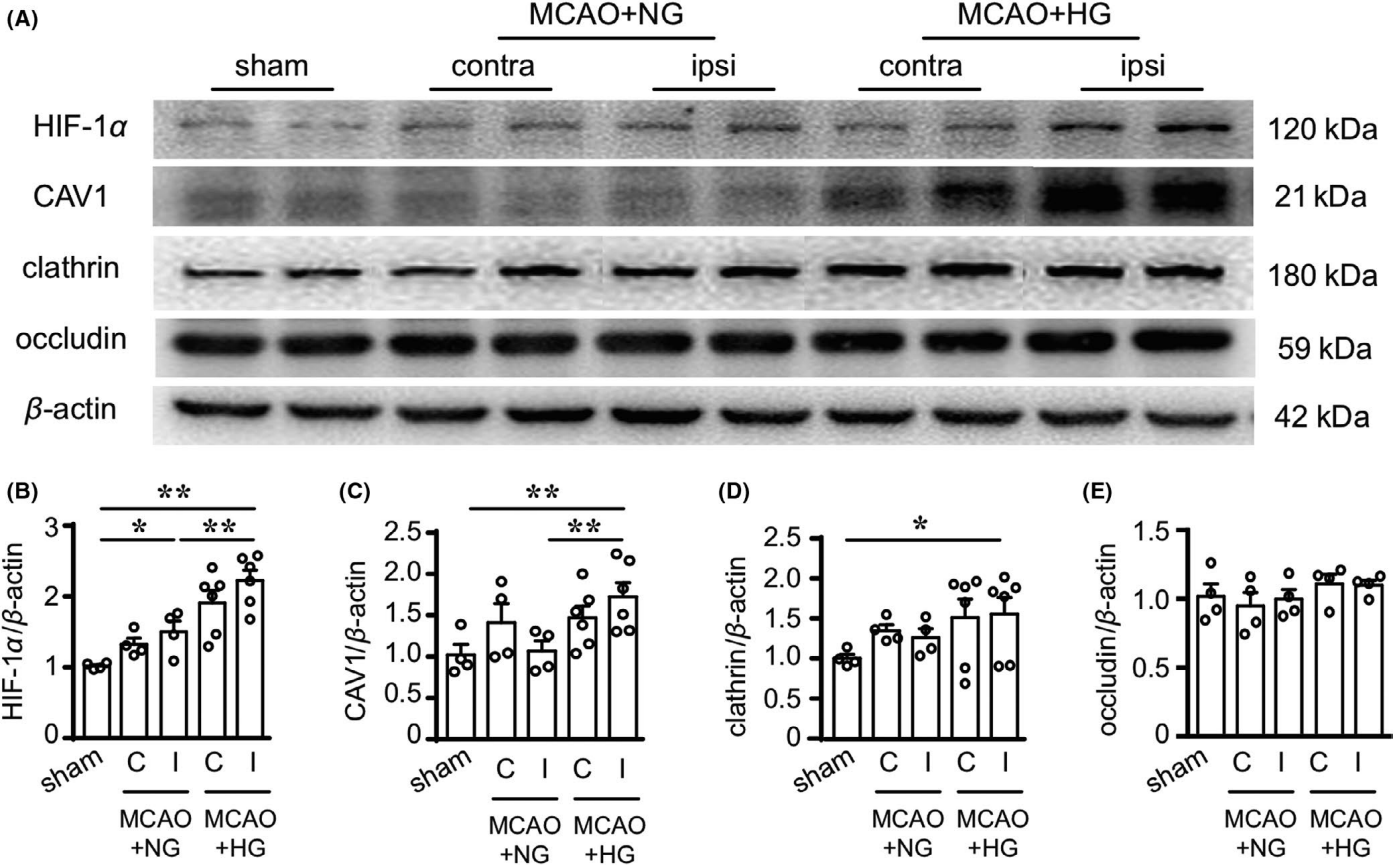
Hyperglycemia upregulates HIF‐1α and endocytosis‐related proteins after mild ischemia/reperfusion brain injury. Protein expression of HIF‐1α, CAV1, clathrin, occludin, and β‐actin was measured in sham‐operated brains or in the contralateral (“C”) and ipsilateral (“I”) striatum of NG and HG brains 4 h after 20‐min MCAO by Western blotting. (A) Representative blots. (B‐E) Summarized quantification data. Data are mean ±SEM. n = 4 for sham and MCAO‐NG groups. n = 6 for MCAO‐HG group. *P < 0.05, **P < 0.01

Mammalian cells utilize multiple endocytic pathways at microscale, such as clathrin‐mediated endocytosis, caveolae‐mediated endocytosis, and other clathrin‐ and caveolae‐independent pathways. Recent studies suggest that clathrin can interact with HIF‐1 and promote angiogenesis under hypoxic conditions, for example, during tumorigenesis.^43^ Furthermore, transcription of CAV1 in response to hypoxic conditions could be directly regulated by HIF‐1.^44^ Based on this prior knowledge, we hypothesized that HG‐induced increase of endothelial vesicles are related to caveolin‐ or clathrin‐mediated endocytosis, considering the diameter of these vesicles. To this end, we examined the expression levels of HIF‐1α, CAV1 and clathrin after MCAO and 4 h of reperfusion in HG and NG mice. HIF‐1α was significantly upregulated in the ipsilateral striatum after I/R injury compared to sham controls, and was further upregulated in the HG group compared to NG mice (Figure 5A,B). Similarly, the expression levels of CAV1 and clathrin were significantly elevated in the ipsilateral striatum of the HG group compared to sham group (Figure 5C,D). In contrast with the enhanced transcytosis signaling, the tight junction protein occluding showed no significant changes among all groups (Figure 5A,E), indicating comparable paracellular permeability of the BBB at this injury stage. Together, these results suggest that the increased endothelial vesicles observed in the HG brain after ischemic stroke resulted from caveolin‐ and clathrin‐mediated endocytosis pathways.

## 4 DISCUSSION

CNS endothelial cells are distinguished from peripheral vascular endothelial cells by two characteristics: 1) A unique tightly connected structure between endothelial cells that prevents water‐soluble molecules from entering the brain parenchyma from the circulatory system; and 2) extremely low levels of intracellular vesicles that is often thought to limit transcellular transport of substances across the endothelium.^45^, ^46^, ^47^ A recent study by Haley et al. reported that in obese mice with severely increased BBB permeability 4 h after stroke,^21^ no disruption of the intercellular junctional structure was evident. The results of our study are consistent with the findings of that paper, indicating that in response to a mild brain injury, there are changes within the BMECs, contributing to in increased BBB permeability. Haley et al. also showed that the number of endothelial cell vesicles increased after MCAO in obese ob/ob mice compared to control ob/‐ mice with a concomitant increase of BBB permeability to horseradish peroxidase (HRP) and IgG, suggesting that the increase in endothelial cell vesicles after cerebral ischemia is a major reason accounting for the early BBB hyperpermeability.^27^, ^48^, ^49^ Obesity and hyperglycemia are both vascular risk factors, and the impact of high blood glucose on the number of vesicles in BMECs has not been addressed previously.

Blood–brain barrier disruption is the key pathophysiological basis for HT after stroke,^7^, ^17^ in the present study, two‐ and three‐dimensional electron microscopy revealed that hyperglycemia increased intracellular vesicles and enhanced endocytosis pathways in BMECs, contributing to increased BBB permeability after mild cerebral I/R injury, and concurrently, the risk of HT becomes more significant. The increase of endothelial vesicle numbers was the most prominent in the NVU in the post‐MCAO striatum, which harbored the most severe BBB breakdown. No alteration of tight junction structure was detected by electron microscopy after MCAO in either NG or HG group. Notably, we also observed the compression of microvessels and swelling of surrounding astrocytes in the ipsilateral NVU under HG condition. I/R brain injury with the comorbid condition of high blood glucose may lead to a vicious cycle of ischemia–edema and exacerbate ischemia–edema in local tissues. A further examination on the related endocytosis pathways revealed the involvement of HIF‐1α. Therefore, brain I/R under HG condition may trigger tissue edema, which further aggravates local hypoxia and upregulates HIF‐1α, subsequently increasing endocytosis. However, the specific mechanisms warrant future explorations.

We also confirmed that the increased vesicles may result from both caveolin‐ and clathrin‐mediated endocytosis pathways. Diabetes elevates the expression of HIF‐1α and vascular endothelial growth factor (VEGF) in the ischemic cerebral microvasculature,^38^, ^50^, ^51^ which is accompanied by the disruption of the BBB, increased infarct volume, severe edema formation, and exacerbated neurological deficits. Specific inhibition of endothelial HIF‐1α could partially reverse the damaging effects of diabetes on cerebrovascular injury, suggesting that HIF‐1α activation is one important mechanism underlying increased BBB permeability.^38^ Our results support the idea that high blood glucose further upregulates HIF‐1α expression to exacerbate BBB impairment after ischemia, possibly by promoting clathrin‐ and caveolin‐mediated endocytosis and transcellular transport in BMECs. However, more evidences of relationship between HIF‐1α and CAV1 or clathrin need to be further studied in the future.

Endothelial cells rapidly respond at the early stage of ischemic stroke by altering their transcellular ratio, and peripheral cells maintain BBB stability by secreting inhibitory signals that reduce the number of vesicles and the rate of transcellular transport in BMECs.^52^, ^53^, ^54^, ^55^ Post‐stroke pericyte and astrocyte injury may unlock this important inhibitory mechanism,^56^ which is necessary to maintain low transcytosis within the endothelium. However, whether or not increased transcellular transport provides a beneficial signal to the NVU during the initial stages of stroke injury progression remains to be explored experimentally.

In conclusion, our study not only identified HG‐enhanced endothelial endocytosis after ischemic stroke, but also clarified that such endocytosis was likely mediated by clathrin and caveolin, presumably also involving regulation by HIF‐1α. The ultrastructural evidence revealed the role of endothelial transcytosis in HG‐exacerbated BBB impairment ahead of physical breakdown of junctional structures between BMECs, which may represent a promising therapeutic target to preserve BBB integrity in stroke patients with HG comorbidities.

## CONFLICT OF INTEREST

The authors declare that they have no conflict of interest.

## AUTHOR CONTRIBUTIONS

X.L. and Y.Z. designed the project and supervised the project. X.X. initiated the project. X.X., L.Z., and J.L. performed the EM experiments and analyzed the data. X.X., K.X., and J.W. performed the animal model and neurological score. X.L., Y.Z., J.G., G.W., and X.X. interpreted the results and commented on the manuscript. X.L. and X.X. wrote the manuscript.

## Supporting information

Supplementary Material

Video S1

Video S2

Video S3

## Data Availability

All original data and materials are available upon request.
