# Erratum

**DOI:** 10.1111/cns.13628

**Published:** 2021-02-25

In Xu et al,^1^ the following errors were published on pages 123 and 124.

1. The affiliation details of the first and eighth authors were incorrectly listed. The corrected list of author affiliations are provided below,

**Xiaomin Xu^1,2^ | Liuqi Zhu^1^ | Ke Xue^1^ | Jiayi Liu^1^ | Jian Wang^1^ | Guohua Wang^1^ | Jin‐hua Gu^1^ | Yunfeng Zhang^1^ | Xia Li^1^**

^1^Institute of Special Environmental Medicine and Department of Neurology of Affiliated Hospital, Co‐innovation Center of Neuroregeneration, Nantong University, Nantong, China

^2^Qidong Women's and Children's Health, Qidong, China

2. The keyword, *blood‐brain barrier* was incorrectly listed as *blood, brain barrier*. The corrected keywords are provided below,

**KEYWORDS**

blood‐brain barrier, electron microscopy, endothelial vesicles, hyperglycemia, neurovascular unit.

The online version of this article has been corrected.
