# Supplementary material for: Ultrastructural studies of the neurovascular unit reveal enhanced endothelial transcytosis in hyperglycemia‐enhanced hemorrhagic transformation after stroke
Source: CNS Neurosci Ther. 2021 Jan 1;27(1):123–33. doi: 10.1111/cns.13571 (PMC7804894; doi:10.1111/cns.13571)

Here are " Full unedited blot for figure 5A ". The lanes marked with the red rectangle are the cropped images appeared in the manuscript. (See as below)

Full unedited blot for HIF-1α and β-actin：

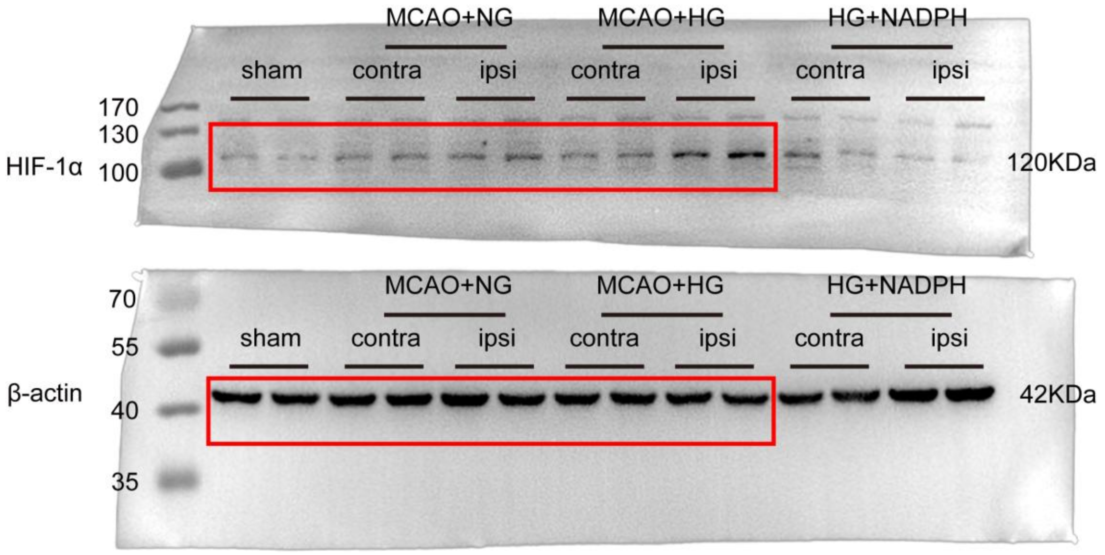

Full unedited blot for CAV1：

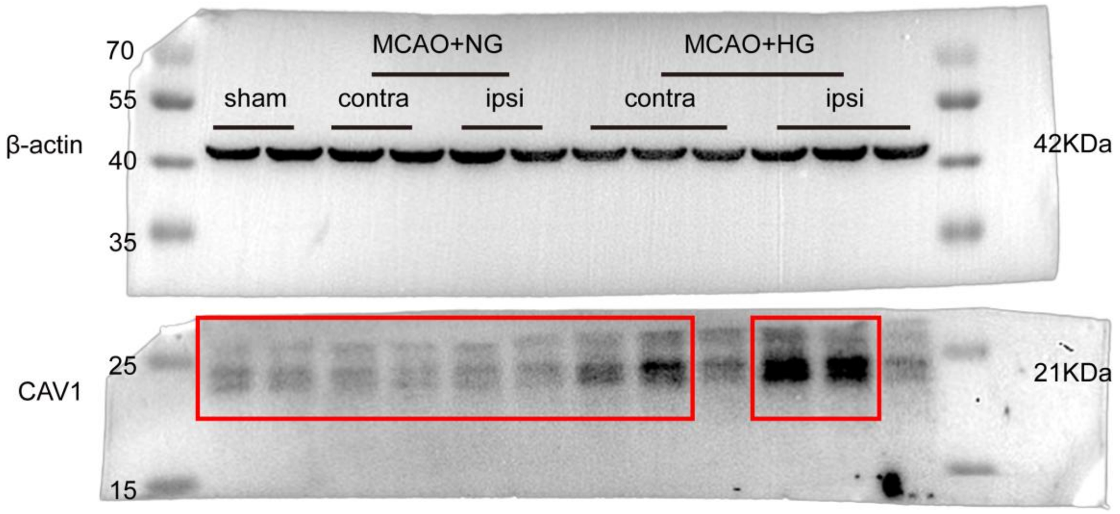

Full unedited blot for clathrin:

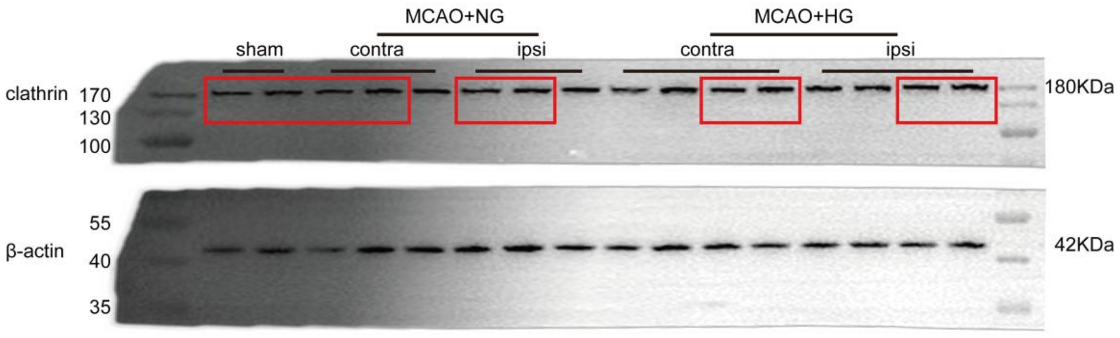

Full unedited blot for occludin:

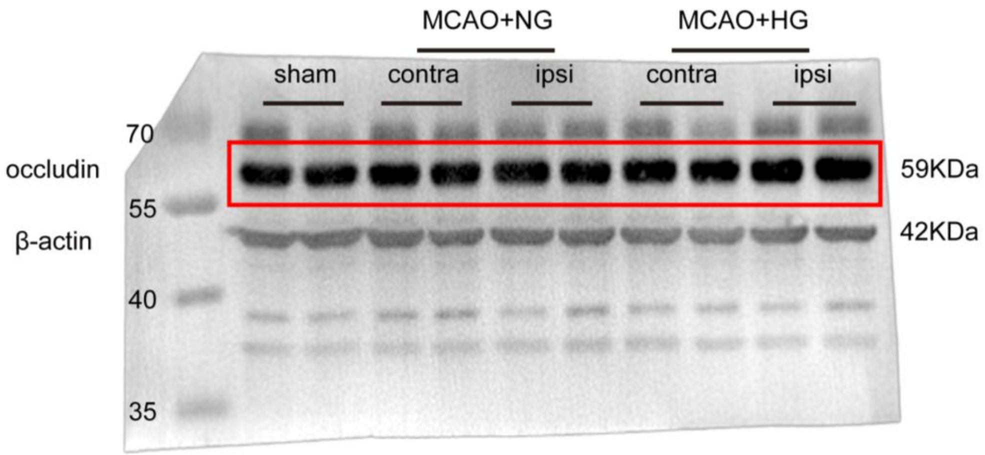

Supplement: Supplementary file 1 — Supplementary Material [file CNS-27-123-s001.pdf]
